# Simultaneous vaccination against seasonal influenza and COVID-19 among the target population in Italy

**DOI:** 10.3389/fpubh.2024.1431963

**Published:** 2024-08-06

**Authors:** Vincenza Sansone, Grazia Miraglia del Giudice, Giorgia Della Polla, Italo Francesco Angelillo

**Affiliations:** Department of Experimental Medicine, University of Campania "Luigi Vanvitelli", Naples, Italy

**Keywords:** COVID-19, influenza, Italy, simultaneous vaccination, survey

## Abstract

**Introduction:**

Annual influenza and COVID-19 vaccinations are effective tools for reducing the disease burden. The goals of the present cross-sectional survey were to investigate attitudes and behaviors toward the simultaneous vaccination against seasonal influenza and COVID-19 and the factors associated.

**Methods:**

Questionnaires were self-administered or researcher-administered between October 2023 and February 2024 in an immunization center in the southern part of Italy.

**Results:**

All 151 subjects eligible for influenza and COVID-19 vaccinations who attended the center agreed to participate. A total of 59.9% of respondents received concurrent seasonal influenza and COVID-19 vaccinations. Those who perceived that the simultaneous vaccination was safer and those who have been infected by SARS-CoV-2 fewer times were more likely to have simultaneously received both vaccinations. Regarding the reasons reported, half of the sample stated that the simultaneous vaccination was safe and that they were adequately informed. This was more likely indicated by the respondents who had received at least four doses of the COVID-19 vaccination. Among those who had not received the simultaneous vaccination, 70.7% and 29.3% had received only seasonal influenza and COVID-19.

**Conclusion:**

Educational health communication campaigns are necessary to improve compliance with simultaneous administration of seasonal influenza and COVID-19 vaccinations and to increase the unsatisfactory coverage.

## 1 Introduction

Annual influenza and COVID-19 vaccinations with high coverage are the primary effective tools for reducing the disease burden related to their morbidity, hospitalizations, and mortality, especially among those that have been identified at an increased risk, such as older people, healthcare workers (HCWs), pregnant women, and people with underlying clinical conditions. In Italy, annual influenza vaccination is recommended and free of charge to several groups, such as people aged ≥60 years, HCWs, individuals with chronic medical conditions and their relatives, pregnant women, and children aged from 6 months to 6 years, with a target coverage of 75% among people aged ≥60 years, HCWs, and individuals with chronic medical conditions (1). However, the uptake is routinely significantly lower than the target, and for those aged ≥65 years, it was only 56.7% in the 2021–2022 influenza season (2). Moreover, in September 2023, an estimated more than 40 million people were eligible for a booster dose of the COVID-19 vaccination, and only approximately 2 million had been vaccinated, despite it being recommended and free of charge for all individuals (3, 4). It is well known that vaccine hesitancy, one of the 10 most serious threats to global health, has a significant impact on vaccinations’ intentions and uptake (5).

The World Health Organization (WHO) recommended the simultaneous vaccination against seasonal influenza and COVID-19 vaccines, considering their public health benefits in terms of acceptance and improvements in the coverage of both and the efficiency of preventive healthcare services (6). Recent studies showed that the concomitant administration of both vaccines elicited immune protection against both viruses, with no safety concerns (7–10). Moreover, although research has focused on the simultaneous vaccination of seasonal influenza with bivalent mRNA COVID-19 (11, 12), to the best of our knowledge, studies conducted in Italy are lacking (13–16). Such data are critically important and required to help decision-making by policymakers and public health professionals to design and develop effective counseling and education interventions aimed at addressing concerns or potential barriers to their adherence and for planning tailored vaccination strategies for simultaneous vaccination among the target population. Therefore, to fill this knowledge gap, the goals of the present cross-sectional survey were to investigate and understand the attitudes and behaviors toward the simultaneous vaccination against seasonal influenza and COVID-19 and the factors that may influence the simultaneous vaccination.

## 2 Materials and methods

### 2.1 Study setting and sample recruitment

This survey took place in a vaccination center in a Teaching Hospital located in the city of Naples, Southern Italy. The sample used all individuals for whom the COVID-19 and annual influenza vaccinations are recommended and available free of charge and who attended the center to receive the COVID-19 or seasonal influenza vaccine between October 2023 and February 2024.

### 2.2 Data collection

Well-trained research investigators, with professional skills in recruiting respondents and knowledge of the topic, approached each subject after the vaccination in the waiting room of the center. Participants were informed about the survey’s objectives and procedures, about data anonymity and confidentiality at all stages, that the survey was answered voluntarily, and that they had the right to withdraw at any time during the survey. Informed verbal consent was obtained from all participants prior to the start of the survey. The research investigators asked each participant to complete the questionnaire and to return it immediately once it was filled. For those who had difficulties in reading or writing, a face-to-face interview was conducted by the research investigators. Participants did not receive compensation or incentive upon questionnaire completion.

### 2.3 Survey instrument

The survey instrument utilized for data collection was adapted from previous similar published surveys conducted by some of us in different groups (17–19). The instrument contained three sections and took an average of 10 min to complete. The first section collected socio-demographic and anamnestic information, including gender, age, marital status, employment, and diagnosis of chronic medical conditions, whether they had been infected by SARS-CoV-2, whether they had received one or more doses of the COVID-19 vaccine, and seasonal influenza vaccination uptake in the previous 2  years. Questions were mainly closed-ended, with simple categorical questions for socio-demographics, yes or no questions, and open-ended. The second section investigated attitudes toward the perceived severity of COVID-19 and influenza diseases, the efficacy, and the safety of the simultaneous vaccination. Respondents reported the extent of their agreement or disagreement with each item administered on a 10-point Likert-type scale ranging from 1 (not at all) to 10 (very much). Participants were asked to indicate their reasons for having received or not received the simultaneous vaccinations from a list of five options, with the possibility to select multiple answers. If respondents did not receive the vaccinations simultaneously, their intention to receive the other one at a different time was measured with an item with three options: “yes,” “no,” and “do not know.” The third section queried the sources of information regarding the simultaneous vaccination and whether they needed additional information.

The questionnaire underwent pilot testing among 15 individuals with the eligible criteria to ensure clarity and comprehensibility. Since no changes were made, the results were included in the analysis.

### 2.4 Statistical analysis

Descriptive statistics were used to present the survey results, including frequencies and proportions for categorical variables, and means, ranges, and standard deviations for continuous variables. Then, the chi-square test or Student’s t-test was used in the bivariate analysis to assess the association between dichotomous and continuous variables and the outcomes of interest. Independent variables with a *p*-value ≤0.25 in bivariate analysis were included in multivariate logistic regression models using a stepwise procedure for variable selection, with a significant level of the *p*-value for the inclusion and elimination of the variables set at 0.2 and 0.4, respectively. The outcomes of interest were the following: having received simultaneously the vaccinations against seasonal influenza and COVID-19 (Model 1) (0 = no; 1 = yes) and having received simultaneously the vaccinations against seasonal influenza and COVID-19 because it was safe and they were adequately informed (0 = no; 1 = yes) (Model 2). The following independent variables have been tested for all outcomes: age in years (<60 = 0; ≥60 = 1), gender (male = 0; female = 1), marital status (unmarried/separated/divorced/widowed = 0; married/cohabitant = 1), baccalaureate/graduate/post-graduate degree (no = 0; yes = 1), having at least one chronic disease (no = 0; yes = 1), having been infected by SARS-CoV-2 (no = 0; once = 1; twice = 2), having received at least four doses of COVID-19 vaccination (no = 0; yes = 1), having received simultaneously the vaccinations against seasonal influenza and COVID-19 in the past (no = 0; yes = 1), having acquired information from scientific societies or journals or meetings (no = 0; yes = 1), and need of additional information (no = 0; yes = 1). The independent variables believed that both diseases were severe (0–9 = 0; 10 = 1), had high concern about getting both diseases (0–9 = 0; 10 = 1), and believed that the simultaneous vaccinations against seasonal influenza and COVID-19 was safe (continuous) have been tested in Model 1. Odds ratios (ORs) and 95% confidence intervals (CIs) were calculated to assess the strength and direction of associations between independent variables and outcomes of interest in the multivariate regression models. A two-sided *p*-value of 0.05 or less was considered statistically significant. All statistical analyses were performed using the STATA 18 software.

## 3 Results

All 151 eligible subjects were enrolled in the survey, yielding a 100% response rate. Table 1 depicts the general demographic data, professional, and anamnestic characteristics of the study population. Most respondents were male, with a mean age of 51.7 years. A total of 41.2% had a post-graduate degree, almost half were HCWs, 40.9% had at least one chronic disease, two-thirds had been infected with SARS-CoV-2 (66%), and more than half after the third dose (51.5%), and only 7.6% and 12.5% self-reported having received vaccinations against seasonal influenza and COVID-19 in 2021 and 2022, respectively.

**Table 1:** Main socio-demographic, professional, and anamnestic characteristics of the sample.

Characteristics	*N*	%
Age, in years	51.7 ± 16.4 (22–77)*	
<60	80	53.3
≥60	70	46.7
Gender		
Male	78	52.4
Female	71	47.6
Marital status		
Married/cohabitant	76	50.3
Unmarried/separated/divorced/widowed	75	49.7
Educational level		
High school degree	32	21.6
Baccalaureate/graduate degree	55	37.2
Post-graduate degree	61	41.2
Employment status		
Unemployed	26	17.4
Employed	123	82.6
Having at least one chronic disease		
No	88	59.1
Yes	61	40.9
Having been infected by SARS-CoV-2		
No	51	34
Yes	99	66
Once	81	82.6
Twice	17	17.4
Time of SARS-CoV-2 infection		
Before vaccination	19	19.2
After the first dose	7	7.1
After the second dose	23	23.2
After the third dose	51	51.5
After the fourth or fifth dose	2	2
Number of COVID-19 vaccination doses received		
1–2	8	5.4
3	70	47.3
4	62	41.9
5	8	5.4
Vaccinations received in 2021		
COVID-19	51	35.4
Seasonal influenza	2	1.4
COVID-19 and seasonal influenza at different times	80	55.6
Simultaneously administered	11	7.6
Vaccinations received in 2022		
COVID-19	41	30.2
Seasonal influenza	6	4.4
COVID-19 and seasonal influenza at different times	72	52.9
Simultaneously administered	17	12.5

Number for each item may not add up to the total number of the study population due to missing values. * Mean ± standard deviation (range).

The results regarding the attitudes toward COVID-19 and influenza diseases and their vaccinations, measured on a 10-point Likert-type scale, showed that participants considered COVID-19 to be more severe than influenza, with mean values of 7.5 and 6.3, respectively. The belief that both diseases were very severe, with values of 10, was indicated by only 8% of the sample. Although the concern of being infected was low, respondents exhibited greater apprehension regarding COVID-19 than influenza, with mean values of 6.3 and 5.5, respectively. The simultaneous vaccination against seasonal influenza and COVID-19 was perceived to be useful and safe, with mean values of 8.3 and 8.

Among all respondents, only 4 out of 151 had already received the seasonal influenza vaccination. Among the remaining 147 participants, vaccinations were simultaneously administered to 88 of them (59.9%). Table 2 displays the results from the multivariate logistic regression models investigating the independent factors associated with the two outcomes of interest. Respondents who perceived safe the simultaneous vaccination against seasonal influenza and COVID-19 (OR = 1.52; 95% CI = 1.22–1.91) and those who had been infected by SARS-CoV-2 fewer times (OR = 0.51; 95% CI = 0.26–0.96) were significantly more likely to receive both vaccinations simultaneously (Model 1).

**Table 2:** Results of the multivariate logistic regression analysis showing the factors associated with the outcomes of interest.

Variable	OR	SE	95% CI	*p*
Model 1. Having received simultaneously the vaccinations against seasonal influenza and COVID-19 Log likelihood = −69.98, χ^2^ = 28.02 (4 df), *p* < 0.0001				
Believing that the simultaneous vaccination is safe	1.52	0.17	1.22–1.91	<0.001
Having been infected by SARS-CoV-2 fewer times	0.51	0.16	0.26–0.96	0.038
Having received at least four doses of COVID-19 vaccination	1.83	0.75	0.82–4.11	0.141
Married/cohabitant	1.47	0.61	0.66–3.29	0.346
Model 2. Having received simultaneously the vaccinations against seasonal influenza and COVID-19 because it was safe and they were adequately informed Log likelihood = −93.45, χ^2^ = 11.33 (4 df), *p* = 0.023				
Having received at least four doses of COVID-19 vaccination	2.05	0.72	1.03–4.09	0.042
Having been infected by SARS-CoV-2 fewer times	0.68	0.19	0.39–1.18	0.175
Males	0.65	0.23	0.32–1.31	0.232
Having acquired information from scientific societies or journals or meetings	1.45	0.52	0.71–2.94	0.306

Belief in the safety of simultaneous vaccinations against seasonal influenza and COVID-19 (59.8%) was the most common reason for deciding to simultaneously receive both, followed by not having to attend the immunization center twice (50.6%) and having adequate information (43.7%). The multivariate logistic regression analysis showed that only one variable was significantly associated with having received both vaccinations simultaneously because it was safe and because they were adequately informed (49%). These reasons were more likely to be indicated by those who had received at least four doses of COVID-19 vaccination (OR = 2.05; 95% CI = 1.03–4.09) (Model 2). Preferring to get the vaccinations at different times (42.4%), having never received two vaccinations at the same time (17%), and being concerned about the safety of the simultaneous vaccinations against seasonal influenza and COVID-19 (11.8%) were the main reasons for not having received both vaccinations simultaneously.

Among the 59 respondents who had not received the vaccinations against seasonal influenza and COVID-19 simultaneously, 70.7% and 29.3% had received only seasonal influenza and COVID-19, respectively. An additional question was about their willingness to be vaccinated against the other disease, with 47.4% were unsure, 28% did not intend to receive it, and only 24.6% were willing to receive it.

The vast majority of the respondents had acquired information about the simultaneous vaccination against seasonal influenza and COVID-19 (87.3%). Scientific journals were the most common source (26.7%), followed by the Internet (24.4%) and mass media (22.9%). Only one-fifth (20.8%) were interested in obtaining additional information.

## 4 Discussion

The present survey provides insight into the simultaneous vaccination against seasonal influenza and COVID-19 and the linked factors, in the post-pandemic phase of COVID-19 among eligible populations in Italy. The information gathered in this investigation provides contributions to the existing literature that should be useful for public health interventions and strategies.

First, less than two-thirds of the respondents (59.9%) had received both vaccinations simultaneously. This result is very similar to that reported among a sample of HCWs in Italy (60%) (20), but considerably higher than what has been previously observed in the United States with 43% (21), 36.5% (22), and 11.1% (12). Moreover, it has been described that administering these vaccinations simultaneously leads to a potential increase in the uptake of COVID-19, primarily because many individuals had received the vaccine against seasonal influenza in previous seasons (23). Among those who did not accept the simultaneous vaccination, two-thirds had been vaccinated against seasonal influenza, and only less than one-third had the COVID-19 vaccination. It is a key point, therefore, to adopt strategies to increase adherence to simultaneous vaccination among those who already get the seasonal influenza vaccination. Nevertheless, a survey conducted by some of us showed that two-thirds were willing to accept the simultaneous vaccination (24). This may be because COVID-19 is no longer a global health emergency, resulting in a consequent lower concern about this disease and a decrease in simultaneous vaccination uptake.

Second, a variety of reasons have been indicated by the sample for their decision to receive or not receive simultaneous vaccinations against seasonal influenza and COVID-19. Belief in the safety of simultaneous vaccinations was the most reported reason. This finding is consistent with previous literature in diverse populations, which revealed that those who intend to get vaccinated against COVID-19 were less likely to believe that the vaccinations were unsafe and also had a more positive attitude toward them (25–28). Moreover, the findings showed that the most frequently cited reasons by the respondents for not having received both vaccinations simultaneously were preferring to get them at different times, having never received two vaccinations at the same time, and safety concerns. The striking finding regarding the concern follows previous literature, also regarding other vaccinations, as vaccine safety has been cited as a reason for non-vaccination (29–31). This finding is still of great relevance from a public health point of view and underscores the importance of addressing the reasons behind low simultaneous uptake and clearing up any misconceptions about the vaccine. By targeting these issues, public health workers and policymakers can work to increase uptake and reduce the burden of diseases.

Third, the results of the multivariate logistic regression analysis revealed interesting and significant associations between the two measured outcomes of interest and the various factors. In regard to the first outcome, respondents who perceived that the simultaneous vaccination was safe were more likely to have received it. This finding confirmed what has been observed in previous research, showing that respondents’ beliefs in the safety and effectiveness of COVID-19 and influenza vaccinations were significant predictors of improving vaccination uptake for COVID-19 (32, 33). Moreover, the positive relationship between fewer times of infection with SARS-CoV-2 and the simultaneous vaccination may be because respondents probably perceive themselves to be more susceptible to getting COVID-19, and this might have encouraged them to protect (34, 35). Regarding the second outcome, the survey showed a positive and statistically significant relationship between having already received at least four doses of the COVID-19 vaccine and the simultaneous vaccination because they believed that it was safe and that they had adequate information. The previous experience with vaccination might have led to this appropriate decision to pay more attention to their health status. This supports the literature that reports having already received vaccinations as an important factor influencing vaccine acceptance against these and other diseases (36–38). Interestingly, scientific sources of information, such as societies, journals, and meetings, had, although not statistically significant, a positive effect on having received the vaccination simultaneously because they believed that it was safe and because they were confident that they had adequate information. In other words, these sources have the potential to influence vaccination decisions and play a key role in improving simultaneous vaccination uptake and positive intention. The association between the use of scientific sources and the vaccinations’ knowledge and attitudes and the likelihood for the population of receiving or for HCWs recommending vaccines to patients is evident in the findings from prior studies conducted in Italy and in other countries, where having received information from these sources is a significant positive and crucial predictor for vaccination initiation (39–42). Therefore, the use of these sources with accurate information is key to improving vaccination rates. Furthermore, the second source of information was the Internet, which is concerning because several previous surveys have shown that those who received information about the vaccines from this source were less likely to be vaccinated and more likely to be hesitant for themselves or their child (43–46).

A number of potential methodological limitations inherent to any similar survey should be acknowledged in interpreting the present findings. First, this used a cross-sectional design; therefore, no causal relationships between the predictors and the different outcomes of interest can be established. Second, the recruitment was conducted only in a vaccination center in a geographic area, and thus the sample may not adequately represent the whole population in Italy, and the findings may not be generalizable. Third, the limited sample size gives results that may not be sufficiently powered to detect a difference between the groups, leading to results that may lack reliability and generalizability. Fourth, the data were collected through self-reporting on vaccine status for up to 2 years, acknowledging the potential for recall bias, and there was no verification of the answers with documentation. Fifth, the attitudes may have been influenced by social desirability bias, with respondents giving favorable comments that led to an overestimation of their intention to have the second vaccine at a different time. However, given the fact that the questionnaire was anonymous, it is plausible that this bias should be limited. Despite these limitations, this survey provides a valuable representation with important implications for building health policy strategies.

To conclude, this survey provides useful information and underscores the critical need for the implementation of targeted evidence-based educational interventions and health communication campaigns with timely and accurate information to enhance simultaneous vaccination against seasonal influenza and COVID-19. Interventions are necessary to improve compliance with the simultaneous administration of these vaccines to reduce the use of resources and increase the very unsatisfactory vaccination coverage.

## Data availability statement

The raw data supporting the conclusions of this article will be made available by the authors, without undue reservation.

## Ethics statement

Ethical approval was not required for the studies involving humans because the survey was conducted according to the guidelines of the Declaration of Helsinki. Ethical review and approval were not required for this survey since the collection of this information is part of the routine health surveillance activities of the Teaching Hospital of the University of Campania “Luigi Vanvitelli.” The studies were conducted in accordance with the local legislation and institutional requirements. The participants provided their verbal informed consent to participate in this study.

## Author contributions

VS: Conceptualization, Data curation, Formal analysis, Writing – original draft. GM: Conceptualization, Data curation, Formal analysis, Writing – original draft. GD: Conceptualization, Data curation, Formal analysis, Writing – original draft. IA: Conceptualization, Data curation, Formal analysis, Funding acquisition, Project administration, Supervision, Validation, Writing – review & editing.
